# Skates and rays (Elasmobranchii, Batomorphii) from the Eocene La Meseta and Submeseta formations, Seymour Island, Antarctica

**DOI:** 10.1080/08912963.2017.1417403

**Published:** 2018-01-04

**Authors:** Andrea Engelbrecht, Thomas Mörs, Marcelo A. Reguero, Jürgen Kriwet

**Affiliations:** a Department of Palaeontology, University of Vienna, Vienna, Austria; b Department of Palaeobiology, Swedish Museum of Natural History, Stockholm, Sweden; c Division Paleontologia de Vertebrados, Museo de La Plata, La Plata, Argentina

**Keywords:** Batoids, Southern Ocean, Paleogene, Antarctic Peninsula

## Abstract

Eocene deposits of the famous La Meseta Formation of Seymour Island, Antarctic Peninsula, yielded the most diverse Paleogene fossil elasmobranch association of the Southern Hemisphere. In this assemblage, sharks clearly dominate the fauna, whereas batoids are very rare components. Herein, we describe two new taxa of cold water tolerant skates, *Marambioraja leiostemma* gen. et sp. nov., and *Mesetaraja maleficapelli* gen. et sp. nov., two new species of the genus *Raja*, *Raja amphitrita* sp. nov. and *Raja manitaria* sp. nov., as well as remains of warm water adapted myliobatiforms. It is, however, not possible to unambiguously assign these remains either to Myliobatidae or Rhinopteridae, or to any specific genus. Previously reported remains of *Raja*/*Bathyraja* sp. are assigned to the new described species *Raja manitaria* sp. nov. The biogeographic distribution of extant and extinct rays and skates clearly shows that both groups are more widely distributed today than in the past, and additionally seem to have been more diverse in the Northern than the Southern Hemisphere. The occurrence, albeit rare of isolated teeth of skates (Rajidae) and rays (Myliobatidae) in the La Meseta Formation representes a minimum age constraint for their first appearance in the Southern Ocean.

http://www.zoobank.org/urn:lsid:zoobank.org:pub:E507D86C-FFEC-4047-A337-AE77606BB1A6

## Introduction

Batoids, commonly known as rays and skates, represent more than half of all extant chondrichthyan taxa, with approximately 26 families and 633 valid species. In contrast, there are about 516 valid species of sharks in more than 30 families (Last et al. 2016). Batoids differ from sharks in having a mostly dorso-ventrally flattened body, ventrally located gill slits, pectoral fins that are often greatly enlarged and either completely (e.g. stingrays) or partly (e.g. guitarfishes) fused and lack anal, caudal, and dorsal fins (Cappetta 2012; Last et al. 2016). Additionally, living batoids vary in their dimensions ranging from about 25 cm to more than 6 m in total length (Last et al. 2016). Most rays and skates occupy demersal habitats and are benthic or bentho-pelagic. Some members, however, have developed a powerful muscular disc, with enlarged fins for swimming actively in the pelagic zone (e.g. *Manta birostris,* Walbaum 1792). Most of the demersal species are highly specialised and highly endemic, whereas pelagic species are typically wide-ranging. Although most extant batoids are fully marine, some groups, like the Potamotrygonidae, successfully adapted to freshwater habitats (Last et al. 2016).

Rays and skates are in general well defined and have an excellent fossil record with the earliest known representatives occurring in Early Jurassic deposits of Europe and Argentina attributable to rhinobatids like *Asterodermus*, *Belemnobatis* and *Spathobatis* (Cappetta 1987; Cione 1999). Batoids became very diverse at the end of the Mesozoic Era (Maisey 2012), and most modern taxa appear in the Cenozoic. However, despite abundant studies about Cenozoic chondrichthyans, the taxonomic diversity and palaeogeographic and stratigraphic distribution of Cenozoic, especially Paleogene, batoids remains incompletely understood. Eocene batoid remains from high southern latitudes are only known from Seymour Island and most specimens thus far known represent isolated teeth of myliobatids (e.g. Cione et al. 1977; Welton and Zinsmeister 1980). Long (1994) was the first to indicate the presence of rajid remains in the Eocene of Antarctica. However, to date no detailed taxonomic analyses of this material have been conducted. Herein, we present new batoid records from the Eocene of Antarctica, which represent the most diverse southern high-latitude batoid assemblage known. This material also allows us to revise previous batoid records from the Eocene of Antarctica.

## Locality and stratigraphic setting

**Figure 1.**
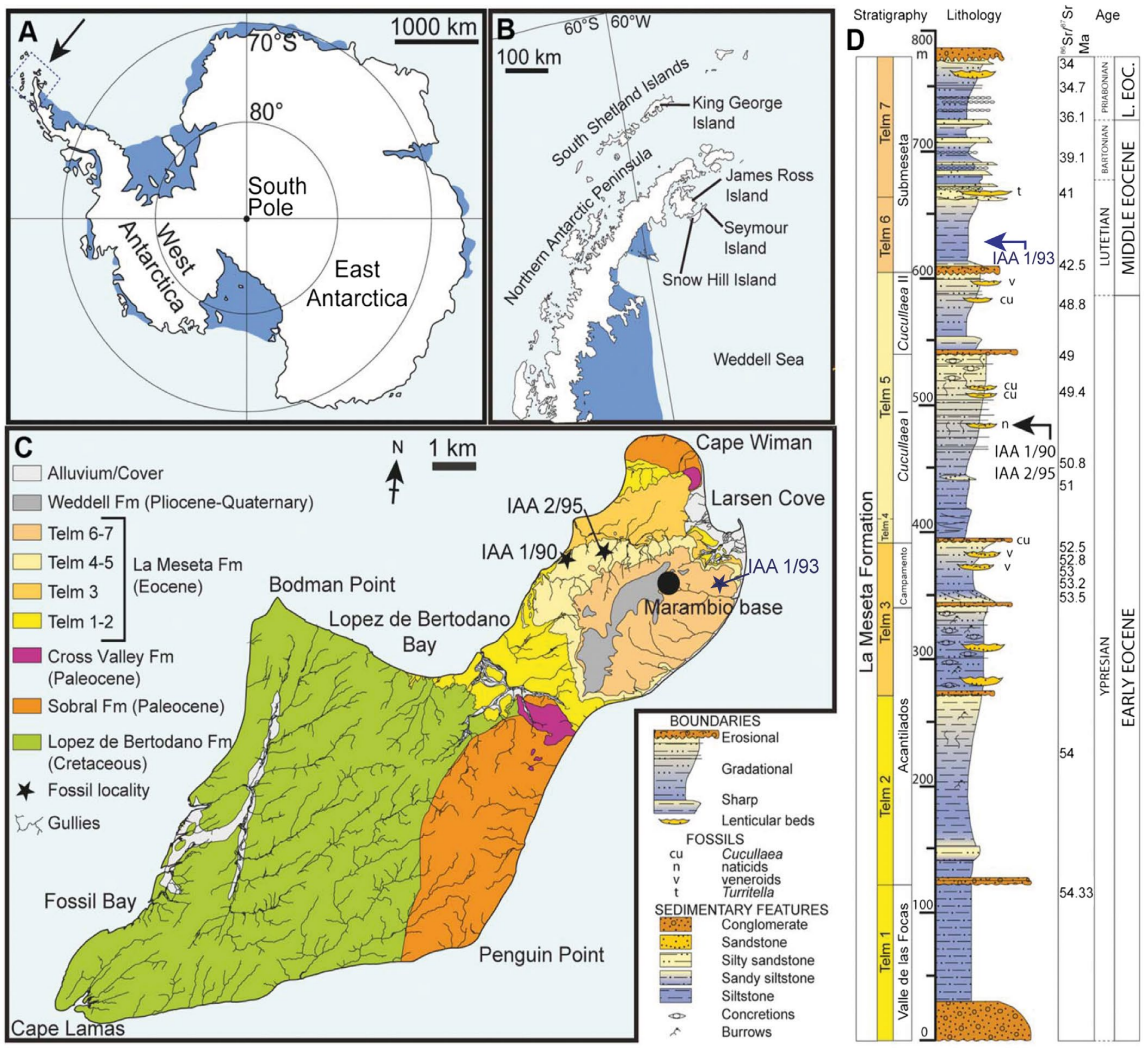
Location and stratigraphy of Seymour Island, Antarctica (A) map of Antarctica, showing the position of the Antarctic Peninsula; (B) map of the Antarctic Peninsula, showing the location of Seymour Island; (C) Geological map of Seymour Island, showing the outcrop of TELMs 5–6 with the localities IAA 1/90, IAA 2/95 and IAA 1/93 of the Eocene La Meseta Formation; (D) composite measured section trough the La Meseta and Submeseta formations, showing the stratigraphical position of the sampled localities IAA 1/90, IAA 2/95 and IAA 1/93. [175 × 160 mm, planned for whole page width]. Notes: Modified from Schwarzhans et al. (2016).

The material forming the basis of this study was derived from Eocene deposits on Seymour Island. This island is located about 100 km east of the Antarctic Peninsula (64°15′S, 56°45′W) in the Weddell Sea (Figure 1(A–C)). Fossiliferous sediments on Seymour Island belong to two groups that span the Late Cretaceous to the Late Eocene/?earliest Oligocene. These include the lower Marambio Group, composed of the Lopez de Bertodano and Sobral formations and the overlying Seymour Island Group consisting of the Cross Valley, La Meseta, and Submeseta formations (e.g. Zinsmeister 1982; Grande and Chatterjee 1987). The approximately 560 meter thick fossiliferous sediments were originally considered a single formation, the La Meseta Fm., which was named and subdivided by Elliot and Trautman (1982). This formation crops out on the northern third of Seymour Island and it consists of poorly unconsolidated mudstones and sandstones with interbedded shell-rich conglomerate filing of a 7 km wide incised valley system (Marenssi 1995; Marenssi et al. 1998a, 1998b). Sediments were deposited in deltaic, estuarine and shallow marine settings.

Sadler (1988) subdivided the La Meseta Formation into seven units that he named TELMs 1 to 7. The sequence was later divided into six erosional units, called allomembers (from base to top: Valle de Las Focas, Acantilados I, Acantilados II, Campamento, *Cucullaea* I and *Cucullaea* II, Submeseta) (Montes et al. 2013). Montes et al. (2013) differentiated the uppermost Submeseta Allomember of the La Meseta Fm. (Marenssi et al. 1998a, 1998b), which is equivalent to TELMs 6 and 7 of Sadler (1988) from the La Meseta Formation and considered it part as Submeseta Fm.

The batoid remains come from four different allomembers (Acantilados, *Cucullaea* I, *Cucullaea* II and Submeseta) corresponding to four TELMs (TELM 2, TELM 4, TELM 5 and TELM 6) of the La Meseta and Submeseta formations, which are Ypresian and Lutetian in age (Figure 1(C–D)). According to Marenssi et al. (1998b) the Acantilados Allomember represents a depositional area in a prodelta of a bay head delta or a low energy mid-estuary setting within the incised valley. The *Cucullaea* I Allomember has a maximum thickness of 90 m and crops out all around the foothills of the meseta. It is composed of shelly channel infills (Marenssi et al. 1998a). The *Cucullaea* II Allomember has the same lithology and sedimentary environments as the *Cucullaea* I Allomember but a maximum thickness of only 50 m. The lithology and the depositional environment of the Submeseta allomember, is similar to those of the *Cucullaea* I and *Cucullaea* II Allomembers consisting of laminated fine-grained sandstones and silty clays with interbedded conglomeratic sandstones (Sadler 1988; Marenssi et al. 1998a).

## Material and methods

The material that forms the focus of this study was collected by an Argentine-Swedish field party as a joint project of the Instituto Antártico Argentino (IAA) and the Swedish Polar Research Secretary (SPFS), during three summer campaigns, 2011–2013, on Seymour Island. It consists exclusively of isolated teeth and is deposited in the palaeozoological collections of the Swedish Museum of Natural History (Department of Palaeobiology), with registration numbers prefixed by ‘NRM-PZ P’.

Approximately 1100 kg of dry-screened sediments (mesh sizes 10 mm, 5 mm, 2.5 mm) were bulk sampled in the field for further processing in the laboratory. Of these 1100 kg, about 350 kg of concentrated sediment from localities IAA 2/95 (‘Marsupial Site’), IAA 1/90 (‘Ungulate Site’) and IAA 1/93 were further processed with mesh sizes of 2, 0.5 and 0.1 mm and subsequently sorted for chondrichthyan and other fossil remains. Small batoid teeth were cleaned with Rewoquat®, than mounted on stubs for photography with a scanning electron microscope (SEM, JEOL 6400). Five views of every specimen (occlusal, labial, lingual, lateral, and basal) were recorded. Larger specimens were photographed with a reflex camera, Nikon D5000 or a 3D microscope camera Keyence VHX-1000D and a 3D data-set was reconstructed with Drishti software v. 2.6.3 (Limaye 2012; https://sf.anu.edu.au/Vizlab/drishti/). Terminologies and the systematics scheme used follows Cappetta (2012).

## Systematic palaeontology

Supraclass CHONDRICHTHYES Huxley, 1880

Class ELASMOBRANCHII Bonaparte, 1838

Superorder BATOMORPHII Cappetta, 1980

Order RAJIFORMES Berg, 1937

Family RAJIDAE Bonaparte, 1831

Genus *Raja* Linnaeus, 1758

*Raja manitaria* sp. nov.

(Figure 2)

**Figure 2.**
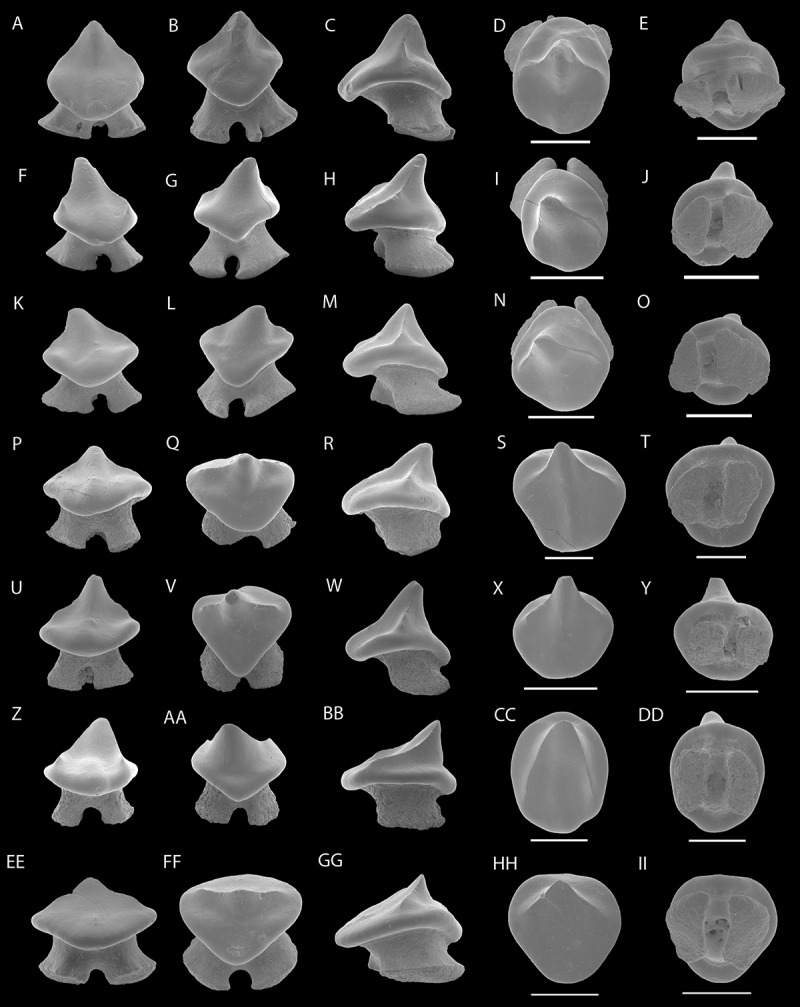
SEM photographs of teeth of *Raja manitaria* sp. nov. male teeth: NRM-PZ P16271, (A), labial; (B), lingual; (C), profile; (D), occlusal; (E), basal views, NRM-PZ P16270, (F), labial; (G), lingual; (H), profile; (I), occlusal; (J), basal views, NRM-PZ P16269, (K), labial; (L), lingual; (M), profile; (N), occlusal; (O), basal views; NRM-PZ P16268, (P), labial; (Q), lingual; (R), profile; (S), occlusal; (T), basal views; NRM-PZ P16267, (U), labial; (V), lingual; (W), profile; (X), profile; (Y), basal views; female teeth: NRM-PZ P16266, (Z) labial; (AA), lingual; (BB), profile; (CC), occlusal, (DD), basal views; NRM-PZ P16265, (EE) labial; (FF), lingual; (GG), profile; (HH), occlusal; (II), basal views. Note: Scale bar equals 1 mm.

**Figure 3.**
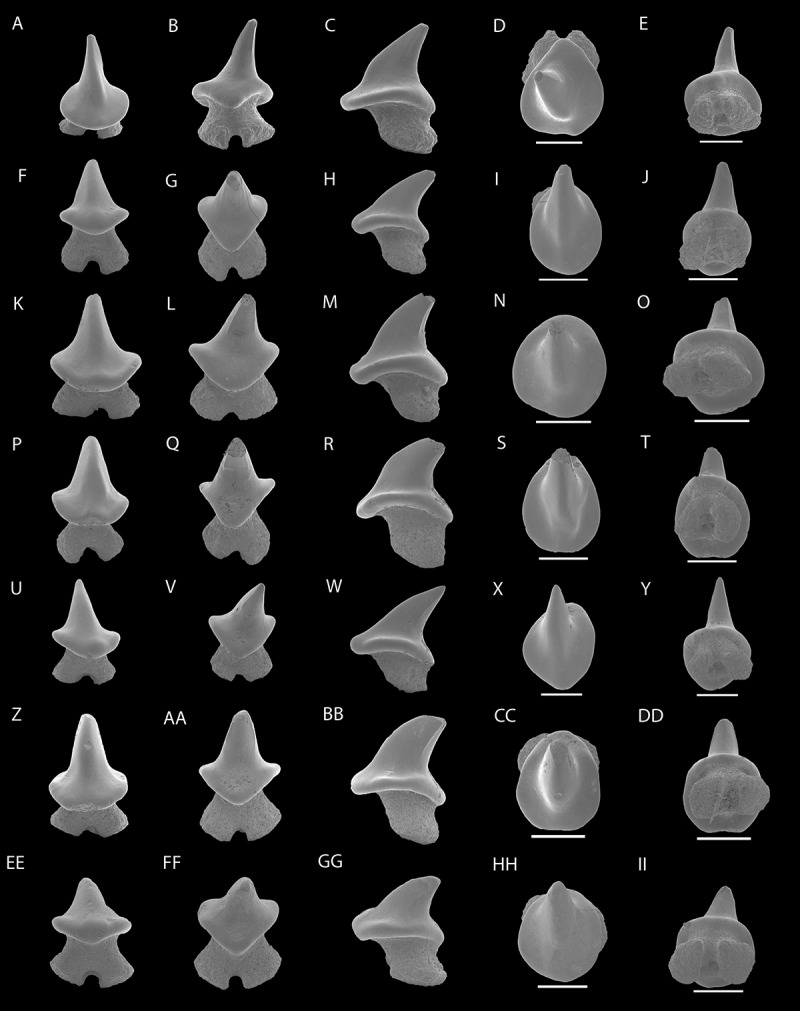
SEM photographs of teeth of *Raja amphitrita* sp. nov. NRM-PZ P16264, (A), labial; (B), lingual; (C), profile; (D), occlusal; (E), basal views, NRM-PZ P16263, (F), labial; (G), lingual; (H), profile; (I), occlusal; (J), basal views, NRM-PZ P16262, (K), labial; (L), lingual; (M), profile; (N), occlusal; (O), basal views; NRM-PZ P16261, (P), labial; (Q), lingual; (R), profile; (S), occlusal; (T), basal views; NRM-PZ P16260, (U), labial; (V), lingual; (W), profile; (X), profile; (Y), basal views; NRM-PZ P16259, (Z) labial; (AA), lingual; (BB), profile; (CC), occlusal, (DD), basal views; NRM-PZ P16258, (EE) labial; (FF), lingual; (GG), profile; (HH), occlusal; (II), basal views. Note: Scale bar equals 1 mm.

*Raja/Bathyraja* sp.: Long 1994, p. 217; Figure 3(A–F)

*Etymology.* The species name is derived from the Greek word ‘manitari’, meaning ‘mushroom’ in allusion to the appearance of the teeth.

*Holotype.* Single male tooth, NRM-PZ P16271 (Figure 2(A–E)).

*Type locality.* Site IAA 2/95 (GPS data: 64°13′58″S; 56°39′06″W), *Cucullaea* I Allomember, TELM 5, La Meseta Fm., Seymour Island, Antarctica, Ypresian, early Eocene.

Type horizon. TELM 5, *Natica*-Horizon, La Meseta Formation.

*Stratigraphic and geographic range.* TELM 5, Ypresian, Early Eocene, IAA 1/90, ‘Ungulate Site’ (GPS data: 64°14′04.67″S; 56°39′56.38″W) and IAA 2/95, ‘Marsupial site’, (GPS data: 64°13′58″S; 56°39′06″W) and TELM 6, Lutetian, Middle Eocene, IAA 1/93 (GPS data: 64°13′51.8″S; 56°35′53.14″W).

*Paratypes.* Two male teeth from site IAA 1/90 (NRM-PZ P16270 and NRM-PZ P16268) and four teeth from site IAA 2/95 (male teeth: NRM-PZ P16269 and NRM-PZ P16267, female teeth: NRM-PZ P16265 to NRM-PZ P16266), TELM 5 (Ypresian, early Eocene), La Meseta Fm.

*Additional material (not figured).* Thirty-six teeth from site IAA 1/90 (NRM-PZ P15782) and 21 teeth from site IAA 2/95 (NRM-PZ P15783), TELM 5 (Ypresian, early Eocene), La Meseta Formation. Five teeth from site IAA 1/93 (NRM-PZ P15784), TELM 6 (Lutetian, middle Eocene), Submeseta Fm.

*Diagnosis*. The new species of *Raja* is defined by the following combination of dental characters: Teeth with a low and triangular cusp that is more or less centred on the crown to slightly shifted towards the distal edge; smooth labial and lingual crown faces; well-developed and irregular cutting edges to cutting edges, which are arched convexly and are continuous through the apex of the principal cusp; mesial and distal rim around the base of the crown on all sides; shallow rounded depression mesial and distal from the triangular cusp in lingual view; the median overhang of the collar is not very prominent, lacking a distinct uvula and apron; labial and lingual crown edges are straight to concave; a low root with well separated and short flaring root lobes; the median furrow is broad and deep.

*Taxonomic comparison*. The teeth of the new taxon *Raja manitria* gen. et sp. nov. differ from teeth of

- *Walteraja exiguia* Siverson and Cappetta, 2011 from the Late Cretaceous of Sweden, in having a lower and broader crown, more or less symmetrical teeth, a narrower root with well separated root lobes and lacking a margino-lingual foramen in the root in the male morphotype, whereas the female morphotype has a lingually displaced crown with a strongly concave crown surface,
- *Raja sudhakari* Prasad and Cappetta, 1993 in having a higher, triangular crown, a well-developed and higher root and a kidney-shaped basal root face,
- *Raja farishi* Case and Cappetta, 1997 in having a rounded to oval occlusal outline, a more slender and slightly lower root,
- ‘*Raja’ louisi* Cappetta, 1972 in having higher teeth, with a high and triangular cusp and a higher root and differs most significantly in lacking a completely vertical labial crown edge,
- *Raja harrisae* Ward, 1984 in having lower teeth with a shorter cusp and lacking a distinct labial cutting edge,
- *Smithraja forestensis* Herman, 1986 in having more gracile teeth with a cuspidate crown and a lower and more slender root,
- *Raja* (*Malacoraja*, *Cruriraja*) *marandati* Adnet, 2006 in having teeth of the male morpohtype with a broader and lower cusp, a narrower root with well separated lobes, and teeth of the female morphotype have a more cuspidate cusp, which is lingually displaced and bears a strongly concave crown surface,
- *Raja* (*Malacoraja*, *Cruriraja*) *michauxi* Adnet, 2006 in having teeth of the male morphotype with a lower and broader principal cusp that is more or less straight and a narrower root, and teeth of the female morphotype with a more cuspidate crown, that have strongly concave crown surfaces and lacking a labial crown ornamentation, booth morphotypes lack a prominent collar,
- *Dipturus casieri* Steurbaut and Herman, 1978 in having a comparable higher crown and slender root lobes, which are separated by a shallower and narrower nutritive groove,
- *Atlantoraja cecilae* Steurbaut and Herman, 1978 in having a lower triangular crown and a narrow and higher root with well separated root lobes,
- *Raja mccollumi* Cicimurri and Knight, 2009 in having a lower and broader cusp, and a comparable higher root,
- extant *Bathyraja* Ishiyama, 1958 in lacking a narrow and elongated principal cusps in all tooth position and in having a low root,
- extant *Amblyraja* Malm, 1877 in lacking a high, slender and narrow principal cusp, a distinct lingual uvula and a broad root with broadly separated root lobes.

*Raja manitaria* gen. et sp. nov. can be distinguished from the other Antarctic taxa described in this study by having teeth with a low triangular central cusp of the male morphotype and a short lingually displaced triangular cusp, with a strongly concave labial crown surface in teeth of the female morphotype. Teeth of both genders possess elongated mesial and distal cutting edges that are slightly sigmoidal in profile view with a lower root compared to the cusp.

*Description.* Teeth of suggested male individuals (Figure 2(A–Y)) are small and slightly wider than 1.0 mm. The teeth are cuspidate, having a low triangular shaped cusp. The labial and lingual crown faces are completely smooth (e.g. Figure 2(A, B, F, G)). The median overhang of the collar by the lingual protuberance is minimal. The cutting edges are very distinct in being continuous across the apex of the principal cusp but not reaching the basal part of the crown (e.g. Figure 2(M, R, W)). The basal part of the crown forms a distinct rounded rim in most specimens (e.g. Figure 2(C, M, R)). In profile view, the median part of the crown is higher than the mesial and distal rim at the crown base and the labial protuberance is short, rounded and straight to slightly basally directed. The lingual protuberance is poorly separated from the lingual crown face at the level of the low triangular crown. The lingual crown edge is straight to slightly concave and the labial crown face is slightly to strongly concave.

In occlusal view, the outline of the crown is rounded to oval shaped. The upper part of the crown is slightly longer than the basal lingual part of the crown. The occlusal crown surface is slightly concave. The low and holaulacorhize root has a deep and broad median furrow, with well-separated and flaring root lobes. The root is devoid of any margino-lingual foramina.

Teeth of supposed female individuals (Figure 2(Z–II)) differ slightly from male teeth in that they have a comparably lower triangular cusp, which is lingually displaced and has a very distinct cutting edges. The mesial and distal cutting edges are arched convexly, rather sigmoid in profile view and continuous through the apex of the cusp (Figure 2(BB)). The labial crown face is strongly concave forming an oval shaped depression and lacks an apron. In labial view, the basal edge of the crown forms a low rounded rim, which extends over the basal labial part of the crown (Figure 2(GG)). The lingual crown face is rather high and the upper edge of the lingual crown face (Figure 2(AA, FF)) is irregular. The apex is rather asymmetrical in profile view, with a very shallow and concave labial face and an almost vertical lingual crown face. The crown surface of female teeth is strongly concavely dented (Figure 2(CC, HH)). This differs significantly from the condition seen on male teeth, where the apex is more erect in profile view with steep and only slightly concave labial and lingual faces. Teeth of both sexes have a rounded to oval outline of the crown in occlusal view and the apex of the crown does not overhang the basal part of the crown.

The root is slightly lingually displaced with a low, almost vertical labial root face. Laterally, shelf-like ridges are present on the root lobes when not abraded.

*Remarks.* Teeth of *Raja manitaria* sp. nov. are similar to previously described specimens that Long (1994) identified as teeth of the probable oldest representantive of *Bathyraja*. Teeth of female *Raja manitaria* sp. nov. are most similar to the specimens figured by Long (1994, Figure 3(E–F)) that he showed in occlusal view. The here described male morph shares characteristics with the other specimens figured by Long (1994, Figure 3(C–D)) such as a low median crown, the characteristic median bulge on the labial crown face, the elongated cutting edges ending at the prominent basal rim of the crown, and rather short but well separated root lobes. We therefore include the five specimens, mentioned and figured by Long (1994) due to the strong resemblance to our newly described species *Raja manitaria* sp. nov.

Although, the new species shares some tooth crown characteristics with teeth of the extant *Bathyraja albumaculata* Norman 1937, they can easily be distinguished by the differently shaped root, and the elongated and asymmetrical crown. Furthermore, in occlusal view the crown apex extends over the lingual crown boarder in *Bathyraja albumacualta*.

The morphological variation in our sample is interpreted to represent a gyandric or sexual heterodonty (compare Figure 2(C and BB)). Teeth of the male morphotype (Figure 2(A–Y)) of *Raja manitaria* sp. nov. are slightly more common than teeth of supposed female representatives (Figure 2(Z–II)). Furthermore, this taxon is restricted to the Eocene of Antarctica and is the most common rajid represented in the La Meseta and Submeseta formations.

*Raja amphitrita* sp. nov.

(Figure 3)

*Etymology.* The species name is derived from the Greek goddess of the sea, ‘Amphitrite’.

*Holotype.* Anterior tooth, NRM-PZ P16264 (Figure 3(A–E)).

*Type locality.* IAA I/93 (GPS data: 64°13′51.8″S; 56°35′53.14″W), Submeseta I, Submeseta Formation, Seymour Island, Antarctica, Lutetian, Middle Eocene.

Type horizon. TELM 6, Submeseta I Allomember, Submeseta Formation.

*Paratypes.* A single ?antero-lateral tooth from site IAA 1/90 (NRM-PZ P16259) and, five anterior to antero-lateral teeth from site IAA 2/95 (NRM-PZ P16260 to NRM-PZ P16263 and NRM-PZ P16258), TELM 5 (Ypresian, Early Eocene), La Meseta Fm.

*Additional material (not figured).* Sixteen teeth from site IAA 1/90 (NRM-PZ P15785) and 61 teeth from site IAA 2/95 (NRM-PZ P15788), TELM 5 (Ypresian, Early Eocene), La Meseta Fm. A single specimen from site IAA 1/93 (NRM-PZ P15790), TELM 6 (Lutetian, Middle Eocene), Submeseta Fm.

*Stratigraphic and geographic ranges.* TELM 5, Ypresian, Early Eocene, IAA 1/90, ‘Ungulate Site’ (GPS data: 64°14′04.67″S; 56°39′56.38″W) and IAA 2/95, ‘Marsupial site’, (GPS data: 64°13′58″S; 56°39′06″W) and TELM 6, Lutetian, Middle Eocene, IAA 1/93 (GPS data: 64°13′51.8″S; 56°35′53.14″W).

*Diagnosis*. Extinct skate only known by isolated teeth that are characterized by the following combination of dental characters: small, cuspidate teeth; high, symmetrical and triangular crown with smooth labial and lingual crown faces; cusp straight to slightly bent towards the posterior; knob-like structure on basal labial crown face; short cutting edges, which are restricted to the upper part of the cusp; lower part of the crown forms a rounded rim extending from the mesial to the distal side of the tooth; distal crown edge concave and mesial crown edge convex to slightly sigmoidal; no labial apron or lingual uvula; high root with root lobes separated by a rather wide but shallow furrow; basal face of the root lobes convex.

*Taxonomic comparison*. Teeth of the new taxon *Raja amphitrita* sp. nov. differ from teeth of

- *Walteraja exiguia* Siverson and Cappetta, 2001 in having more or less symmetrical teeth, with a higher and more slender principal cusp, a narrower root base and lacking margino-lingual foramina,
- *Raja sudhakari* Prasad and Cappetta, 1993 and *Raja farishi* Case and Cappetta, 1997 in having high cuspidate crowns, a more or less rounded occlusal outline, a high and broader root, with kidney-shaped basal root lobe faces,
- ‘*Raja’ louisi* Cappetta, 1972 in having high cuspidate teeth, with a narrower crown, *Raja harrissae* Ward, 1984 most significantly in having a labial ridge and a more gracile crown,
- the male morphotype of *Raja* (*Malacoraja*, *Cruriraja*) *marandati* Adnet, 2006 and *Raja* (*Malacoraja*, *Cruriraja*) *michauxi* Adnet, 2006 in having rather straight cuspidate teeth, with a slightly broader and lower principal cusp. The female morphotype of the former two taxa differ from *Raja amphitrita* sp. nov. in having a low crown, where the principal cusp is lingually displaced. Both morphotypes differ from *Raja amphitrita* sp. nov. in the very broad root. Additionally, specimens of *Raja amphitrita* sp. nov. lack an labial ornamentation on the crown,
- *Smithraja forestensis* Herman, 1986 in having a high and slender cusp, a lower and more gracile root with massive and elongated root lobes, which are separated by a broad median furrow,
- *Dipturus casieri* Steuerbaut and Herman, 1978 in lacking a distinct uvula and having a high cuspidate cusp with a narrow crown base, and a high and narrow root,
- *Atlantoraja cecilae* Steurbaut and Herman, 1978 in having a narrow cusp, a high root and lacking very wide root lobes,
- the male morphotype of *Raja mccollumi* Cicimurri and Knight, 2009 in having a lower and slightly broader cusp, and a labial cutting edge. The female morphotype of *Raja mccollumi* differs in having a low cusp, which is lingually displaced and both morphotypes differ in having a low root,
- extant *Bathyraja* Ishiyama, 1958 in having a lower principal cusp and a lower root,
- extant *Amblyraja* Malm, 1877 in lacking a distinct lingual uvula.

R*aja amphitrita* sp. nov can be distinguished from the other taxa described in this study by their high and triangular crown that is slightly bent lingually, the short cutting edges, and high root with, lobes separated by a narrow and shallow furrow.

*Description*. The teeth of *Raja amphitrita* sp. nov. are small, measuring slightly more than 1 mm in width. The cusp is high and triangular (e.g. Figure 3(A, P, U)). The height of the crown slightly differs between specimens, which may be an indicator for different sexes. However, the apex is more or less abraded in most teeth due to wear. The labial and lingual crown faces are devoid of any ornamentation (e.g. Figure 3(F, G, Z, AA)). In labial view, the cusps are slightly bent towards the rear in some teeth, but on the majority of teeth the cusp is, completely straight (e.g. Figure 3(C, M, W, BB)). The labial protuberance is very weakly developed. Basally, a more or less pronounced knob-like structure is located on the labial crown face (e.g. Figure 2(EE)). The cutting edges are rather blunt, very short and restricted to the upper portion of the cusp, not reaching the basal part of the crown (e.g. Figure 3M, BB). Labio-basally, a rim-like bulge is developed, which is continuous from the mesial to the distal edge (e.g. Figure 3(M, R, BB)). The lateral edges of the crown form short, almost horizontal heel-like extensions, of which the distal one is usually more elongate than the mesial one.

The cusp is lingually inclined so that the lingual edge of the cusp is strongly concave, whereas the labial edge is convex to slightly sigmoidal in profile view (e.g. Figure 3(C, R)). On most teeth the lingual protuberance is poorly separated from the rest of the lingual crown face (e.g. Figure 3(G, L, FF)). The lower edge of the lingual crown face is triangular. The basal part of the crown is rounded to slightly oval, when viewed occlusally (e.g. Figure 3(I, N, S, HH)).

The root is high and holaulacorhize, with a broad but rather narrow furrow separating the root lobes (e.g. Figure 3(F, P, EE)). The root is narrower under the crown with slightly flaring root lobes. The basal root face is convex and has a single median foramen (e.g. Figure 3(T, DD, II)).

*Remarks.* Teeth of *Raja amphitrita* sp. nov. show no gynandric heterodonty as the herein described teeth of *Raja manitaria* sp. nov. and *Marambioraja leiostemma* gen. et sp. nov. As indicated above, the differences in crown height and width could indicate that female and male morphotypes are present in this sample, but it also points strongly to the natural variation of teeth within a tooth file, where the teeth get slightly lower and broader from anterior to posterior positions. Further, in many cases, extant juvenile males have tooth morphologies, which are similar to that of adult females, which usually change when they reach sexual maturity (e.g. Taniuchi and Shimizu 1993; Kajimura and Trica 1996; Sáez and Lamilla 1997). Because of the very minor dental variations in *Raja amphitrita* sp. nov. we presume that this species exhibits sexual homodonty like the extant *Bathyraja griseocauda*, Norman 1937 (Sáez and Lamilla 2004). However, teeth of extant *Bathyraja* species are too insufficiently known so that it currently is not possible to assign this species unambiguously to *Bathyraja*. Therefore, we assign this species momentarily to the genus *Raja*.

*Marambioraja* gen. et sp. nov.

*Type species. Marambioraja leiostemma* gen. et sp. nov.

*Etymology.* The genus name is derived from the Argentine name for Seymour Island, ‘Isla Marambio’.

*Diagnosis.* Extinct rajid only known by isolated teeth, which are characterized by the following combination of characters; small teeth (approximately 1 mm in width); crown as wide as the root; pyramidal rather low crown, with nearly smooth labial and lingual surface; labial protuberance more pronounced in lateral teeth and slightly pointing downwards; knob-like labial protuberance with flat oval structure centred on the labial face of the protuberance; cutting edges do not reach base of the crown in anterior teeth; smooth occlusal crown face in anterior teeth; lateral teeth have two transversely directed ridges on the occlusal surface; uvula lacking in all tooth positions; basal edge of the lingual crown face slightly triangular; root lobes well separated by a broad and deep furrow; edges of basal root lobe base rounded and some widened at their lower parts; basal root face slightly convex.

*Marambioraja leiostemma* gen. et sp. nov.

(Figure 4)

**Figure 4.**
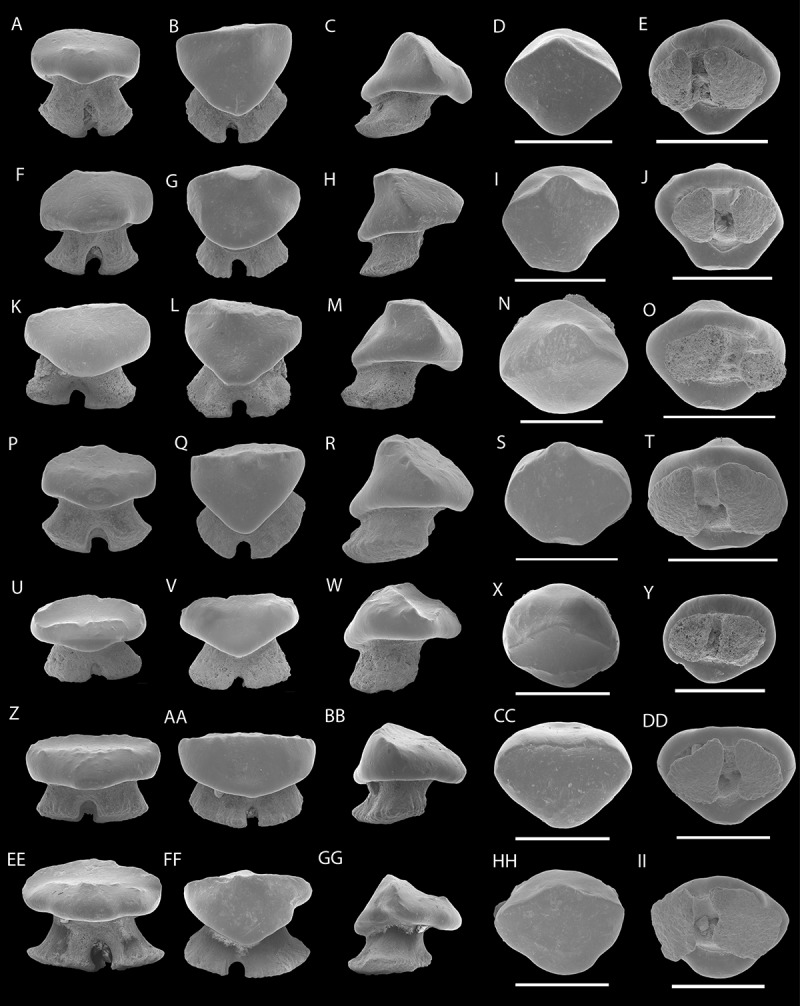
SEM photographs of teeth of *Marambioraja leiostemma* gen. et sp. nov., anterior teeth: NRM-PZ P16257, (A), labial; (B), lingual; (C), profile; (D), occlusal; (E), basal views, NRM-PZ P16256, (F), labial; (G), lingual; (H), profile; (I), occlusal; (J), basal views, NRM-PZ P16255, (K), labial; (L), lingual; (M), profile; (N), occlusal; (O), basal views; NRM-PZ P16254, (P), labial; (Q), lingual; (R), profile; (S), occlusal; (T), basal views; lateral to posterior teeth: NRM-PZ P16253, (U), labial; (V), lingual; (W), profile; (X), profile; (Y), basal views; NRM-PZ P16252, (Z) labial; (AA), lingual; (BB), profile; (CC), occlusal, (DD), basal views; NRM-PZ P16251, (EE) labial; (FF), lingual; (GG), profile; (HH), occlusal; (II), basal views. Note: Scale bar equals 1 mm.

*Etymology.* The species name is derived from ancient Greek word ‘leios’ meaning ‘smooth’ and the Greek word ‘stemma’ for ‘crown’.

*Holotype.* Anterior tooth of male morphotype, NRM-PZ P16257 (Figure 4(A–E)).

*Type locality.* Site IAA 2/95 (GPS data: 64°13′58″S; 56°39′06″W), *Cucullaea* I Allomember, TELM 5, La Meseta Formation, Seymour Island, Antarctica, Ypresian, Early Eocene.

Type horizon. TELM 5, *Natica*-Horizon, La Meseta Formation.

*Paratypes.* Three isolated teeth of the male morphotype from site IAA 2/95 (NRM-PZ P16256 – NRM-PZ P16254), and three isolated teeth of the female morphotype from site IAA 2/95 (NRM-PZ P16251 – NRM-PZ P16253), TELM 5 (Ypresian, Early Eocene), La Meseta Fm.

*Additional material (not figured).* Two isolated teeth from site NRM 1 (NRM-PZ P15791), TELM 4 (Ypresian, early Eocene). Ten isolated teeth from site IAA 1/90 (NRM-PZ P15792) and 17 isolated teeth from site IAA 2/95 (NRM-PZ P15793), TELM 5 (Ypresian, Early Eocene).

*Stratigraphic and geographic Range.* TELM 4; Ypresian, Early Eocene, NRM 1, (GPS data: 64°14.285′S, 56°40.182′W); TELM 5, Ypresian, Early Eocene, IAA 1/90, ‘Ungulate Site’ (GPS data: 64°14′04.67″S; 56°39′56.38″W); IAA 2/95, ‘Marsupial Site’ (GPS data: 64°13′58″S; 56°39′06″W).

*Diagnosis.* As for genus.

*Description.* Anterior teeth of the male morphotype are small, approximately 1 mm in total width (mesio-distally). The crown is slightly wider than the root (mesio-distally) and overhangs the root on all sides (e.g. Figure 4(F)). In labial view, the crown is low and has an oval shaped outline (e.g. Figure 4(A)). The occlusal crown face bears two parallel ridges that are mesio-distally directed, but otherwise devoid of any ornamentation (e.g. Figure 4(P)). The lingual crown face is completely smooth and the lingual protuberance is continuous with the lingual crown face (e.g. Figure 4(G, Q)). Lingually, the upper edge of the crown is more or less straight, whereas the lower part of the crown is triangular (e.g. Figure 4(B)).

In profile view, the lingual crown face is straight to slightly convex, whereas the labial crown face is more labially protruding and convex (e.g. Figure 4(H, R)). The labial protuberance is very distinct, forming a ‘nose-like’ extension (e.g. Figure 4(H)). In occlusal view, the crown is rectangular with concave margino-labial and margino-lingual margins to rounded or even triangular, with a concave anterior margin in teeth of presumably lateral position (e.g. Figure 4(S)).

In basal view, the surface of the labial visor is relatively flat (e.g. Figure 4(J)). The root is more or less as high as the crown or slightly higher. There is a slight median overhang of the collar by the uvula. The root is smooth and no foramina are present. The root lobes are separated by a rather low and narrow furrow (e.g. Figure 4(A, F)). The furrow bears one to two central foramina. The root lobes are basally flared, with the basal edges of the lobes straight to slightly rounded. The basal faces of the root lobes are slightly convex and broadly D-shaped (e.g. Figure 4(J, T)). Additionally, the basal surface of the root lobes are oblique in labial and lingual views.

Anterior teeth of the female morphotype are small (1 mm) and with a crown that is slightly wider than the root. The crown is domed and devoid any ornamentation (e.g. Figure 4(X)). In profile view, the lingual face is slightly concave, whereas the labial crown face is convex (e.g. Figure 4(W, GG)) and is pyramidal in shape. In labial view, the labial protuberance is knob-like, broad, very short and has a rounded basal edge (e.g. Figure 4(W)). It prominently protrudes over the root labially and its rounded tip points slightly basally. In labial view, the cutting edges do not reach the base of the crown. The upper edge of these ridges is not even, and between them, the crown face is slightly concave (e.g. Figure 4(U)). The labial face of the crown is conspicuously concave.

The lingual crown face of both morphotypes is high and a lingual uvula cannot be observed in all tooth positions (e.g. Figure 4(B, AA)). In lingual view, the basal edge of the crown is rounded to slightly triangular (e.g. Figure 4(FF)). The apex of most teeth is abraded but devoid of any ornamentation. The crown is rectangular to pentagonal (e.g. Figure 4(X, CC)). The transverse crest occasionally joins the labial transversal crest marginally (e.g. Figure 4(X)). In basal view, the labial visor forms a curve along the border of the collar.

The root of the female morphotype has more or less the same height as the crown and is obviously holaulacorhize when viewed labially. It is narrower directly under the crown but widens slightly at its basal parts (e.g. Figure 4(Z)). The root lobes are clearly separated from each other by a deep but narrow furrow. The furrow bears one to two small foramina. The basal edge of the root lobes is slightly rounded when viewed labially or lingually. Basally, the root lobes are kidney-shaped and convex (e.g. Figure 4(DD, II)).

*Taxonomic comparison*. Teeth of *Marambioraja leiostemma* gen. et sp. nov. differ from

- the male morphotype of *Walteraja exiguia* in having a low crown, whereas the female morphotype differs in lacking a short triangular principal cusp. Both morphotypes of *Walteraja exiguia* can be distinguished in having a broad root base with not completely separated root lobes and a pair of margino-lingual foramina,
- *Raja sudhakari* in having a rounded to oval shaped crown outline in occlusal view and a higher root. Teeth of the female morphotype of *Marambioraja leiostemma* gen. et sp. nov. differ in having a very low and broad crown mesio-distally,
- *Raja farishi* in having a low crown and lacking a high cusp in both morphotypes,
- *Raja harrisae* in lacking a triangular crown, a labial ridge edge and a basally concave rim at the crown base,
- *Raja (Malacoraja, Cruriraja) marandati* and *Raja (Malacoraja, Cruriraja) michauxi* in having low and rather bulky teeth, with an oval outline in occlusal view in both genders. The female morphotype of *Raja* (*Malacoraja*, *Cruriraja*) michauxi can be further distinguished in having an ornamented labial crown face,
- the female and male morphotypes of *Smithraja forestensis* in having a higher labial and lingual crown face, a more gracile root divided by a deep and narrow furrow and slender root lobes,
- the male morphotye of *Atlantoraja cecilae* in lacking a cuspidate crown and a short and basally very broad root. Teeth of the female morphotype of *Atlantoraja cecilae* differ from that of *Marambioraja leiostemma* gen. et sp. nov. in having a low triangular cusp and lacking two transversally directed ridges on the labial crown surface,
- teeth of *Dipturus casieri* in lacking two transversally directed ridges on the labial crown face in the female and male morphotype and having a cuspidate crown in the male morphotype and a rather broad furrow dividing the root lobes,
- *Raja mccollumi* in having a high cuspidate crown in representatives of the male morphotype and low cuspidate crowns of the female morphotype. Both morphotypes of *Raja mccollumi* lack the characteristic pyramidal shaped crown in male teeth of *Marambioraja leiostemma* gen. et sp. nov. and the two transversal ridges on the labial crown face of the female morphotype,
- extant *Bathyraja* in lacking high cuspidate crown and a high root,
- extant *Amblyraja* in lacking a well separated lingual uvula at the base of the crown.

*Marambioraja leiostemma* gen. et sp. nov differs from all other Antarctic taxa, described herein, in having a low crown, with lateral teeth that possess a nearly closed and oval transversal crest.

*Remarks.* Female teeth of *Marambioraja leiostemma* gen. et sp. nov. differ from corresponding teeth of male morphotypes in having a lower pyramidal-shaped crown, a lower lingual crown surface and two prominent transversal ridges on the labial crown face. As in the other rajid taxa described in this study the morphological variation of representatives of *Marambioraja leiostemma* gen. et sp. nov. is interpreted to represent sexual heterodonty (compare Figure 4(C and BB)). Teeth of this taxon are rather rare components of the batoid fauna compared to the other taxa herein.

*Mesetaraja* gen. nov.

*Type species. Mesetaraja maleficapelli* gen. et sp. nov.

*Etymology.* The generic name is in allusion of the La Meseta Formation from which this taxon derives.

*Diagnosis.* Extinct rajid known only by a single tooth that is characterized by the following combination of dental characteristics: small tooth (approximately 1 mm wide); crown wider than root; crown completely smooth and consists of a labio-lingually broad but mesio-distally narrow cusp with accessory labial and lingual apices that are arranged labio-lingually (central one being the highest); cusp and accessory cusplets give cusp a ragged profile; anterior accessory cusplet slightly hook-shaped; basal crown face asymmetrical and ovate; short, broad and rounded labial apron; distinct lingual uvula absent; basal crown base forms a slightly convex rim around cusp.

*Mesetaraja maleficapelli* gen. et sp. nov.

(Figure 5)

**Figure 5.**
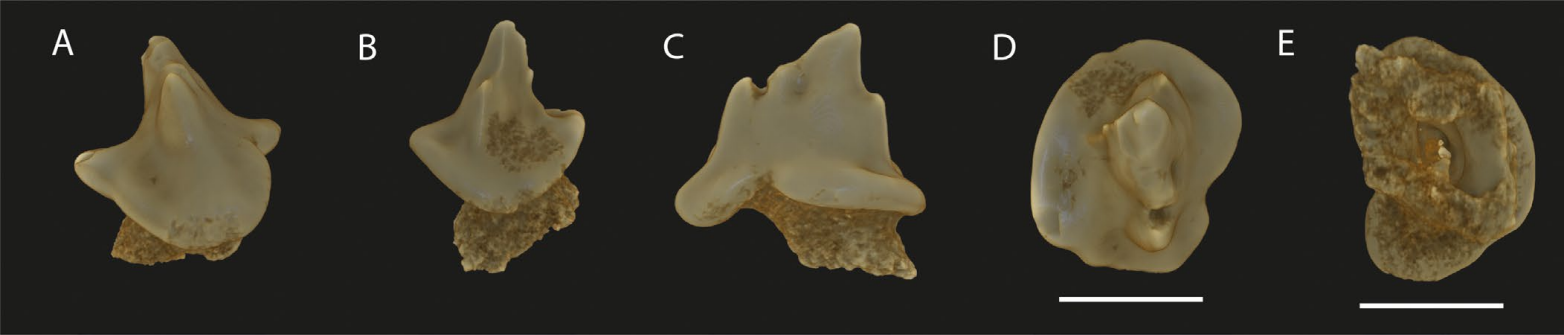
Reconstruction of teeth from a 3D data-set made with Drishti software of *Mesetaraja maleficapelli* gen. et sp. nov. NRM-PZ P16250, (A), labial; (B), lingual; (C), profile; (D), occlusal; (E), basal views. Note: Scale bar equals 1 mm.

*Etymology.* The species name derived from the Latin word ‘malefica’ meaning ‘witch’, and the Latin word ‘capelli’ meaning ‘hat’ due to the resemblance of a witch hat, in profile view.

*Holotype.* Single ?anterior tooth NRM-PZ P16250 (Figure 5).

*Type locality.* Site IAA 2/95 (GPS data: 64°13′58″S; 56°39′06″W), *Cucullaea* I Allomember, TELM 5, La Meseta Formation, Seymour Island, Antarctica, Ypresian, Early Eocene.

*Type horizon.* TELM 5, *Natica*-Horizon, La Meseta Formation.

*Stratigraphic and geographic range.* TELM 5 (Ypresian, Early Eocene): IAA 2/95, ‘Marsupial Site’ (GPS data: 64°13′58″S; 56°39′06″W).

*Diagnosis*. As for genus.

*Description.* This species is known from a single small (1 mm in mesio-distal width) and rather high-crowned tooth. The cusp is very distinct in being high, labio-lingually broad but mesio-distally narrow, and displaced lingually. The apex is inclined slightly lingually and in profile view the labial edge is slightly concave. Conspicuous smaller, accessory cusplets are located labially and lingually to the main cusp. In occlusal view, the smallest accessory cusplet is situated on the lingual side of the crown whereas the highest accessory cusp is adjacent to the smallest one. All cusplets and the main cusp apices show signs of wear. The cutting edge is indiscernible on the labial cusp, but becomes stronger towards the lingually located accessory cusp and runs basally to the base of the lingual accessory cusp.

In occlusal view, the margino-lingual margins are differentially deeply incised, forming a distinct notch on the labial side of the crown. In profile view the labial apron is short, rounded and slightly directed basally. The enameloid of the labial visor is smooth and slightly convex. The tooth lacks a lingual uvula. In occlusal view the basal face of the crown is asymmetrical and slightly oval shaped. At the base of the crown a slightly convex rim is present. The lower part of the root is broken. In basal view, the rather large pulp cavity of the crown is exposed.

*Remarks.* Herman et al. (1994, 1995, 1996) extensively documented the dental morphologies of extant rajid genera and species. The authors characterized one to three species in each genus by their teeth and comparison of *M*. *maleficapelli* gen. et sp. nov. to their figured teeth of extant species revealed no resemblance to any of them. Extinct species including those described herein, *Raja manitaria* sp. nov., *Raja amphitrita* sp. nov., and *Marambioraja leiostemma* gen. et. sp. nov., differ completely in crown morphology (e.g. form of basal rim and presence of additional, accessory cusplets labial and lingual to the main cusp).

The tooth of the new described and unique Antarctic taxon, *Mesetaraja maleficapelli* gen. et sp. nov. thus is not comparable to any described rajid taxon. A malformation of this tooth can be excluded, because it is generally more common in sharks but rarely known in skates (Gudger 1933; Sáez and Lamilla, 2003). Abnormal teeth are recognized as morphological properties that lie outside the range of normal tooth variation, like a twisted crown tip or a deformed root (Becker et al. 2000).We are confident that this tooth can be assigned to a new genus, due to its unique characters described above.

Order Myliobatiformes Compagno 1973

Myliobatiformes gen. indet

(Figure 6)

**Figure 6.**
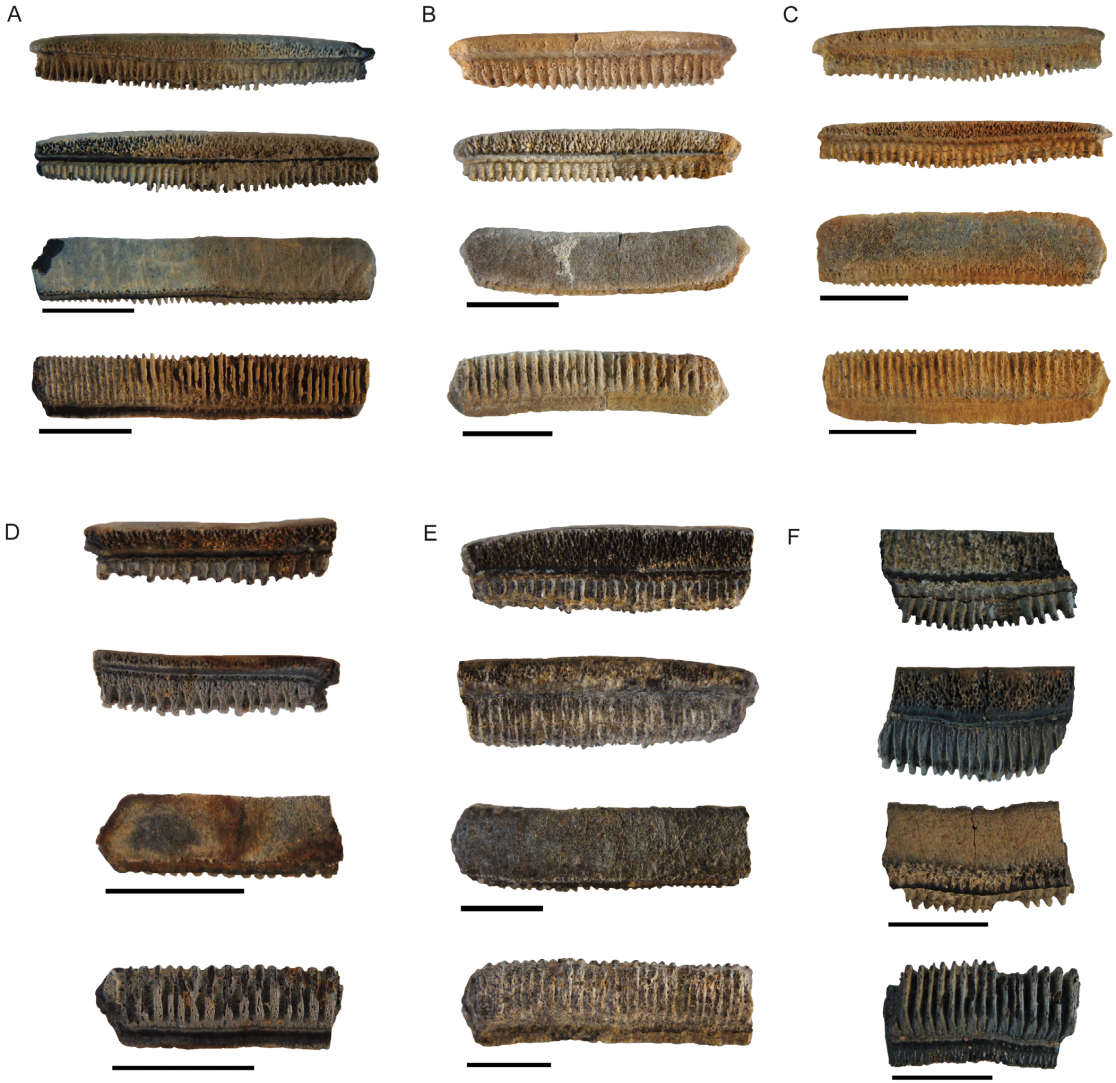
Photographs of *Myliobatis* sp. taken with a reflex camera, morphotype 1, NRM-PZ P16249, (A1), labial, (A2), lingual; (A3), occlusal; (A4), basal views; NRM-PZ P16248, (B1) labial, (B2), lingual; (B3), occlusal; (B4), basal views; NRM-PZ P16247, (C1) labial, (C2), lingual; (C3), occlusal; (C4), basal views; morphotype 2, NRM-PZ P16246 (D1) labial, (D2), lingual; (D3), occlusal; (D4), basal views; NRM- PZ P16245 (E1) labial, (E2), lingual; (E3), occlusal; (E4), basal views; morphotype 3, NRM-PZ P15781, (F1), labial; (F2), lingual; (F3), occlusal; (F4), basal views. Note: Scale bar equals 10 mm.

*Material.* One specimen (NRM-PZ P16249) from IAA 1/11, TELM 4 (Ypresian, Early Eocene); two specimens (NRM-PZ P16248 and NRM-PZ P16248) from NRM 1, TELM 4 (Ypresian, Early Eocene); one specimen (NRM-PZ P16245) from *Cucullaea* intermedia, TELM 4 (Ypresian, Early Eocene); one specimen from *Natica*-Horizon (NRM-PZ P16246), TELM 5 (Ypresian, Early Eocene); and a single specimen (NRM-PZ P15781) from IAA 1/95, TELM 5 (Ypresian, Early Eocene).

*Additional material (not figured)*. A single specimen (NRM-PZ P15824) from *Natica*-Lens, TELM 2 (Ypresian, Early Eocene) and two specimens from Channel Site (NRM-PZ P15825), TELM 2 (Ypresian, Early Eocene).

Five specimens from the Shark basin, *Cucullaea* I (NRM-PZ P15826), TELM 4 (Ypresian, Early Eocene), four specimens from South Rim of *Cucullaea* I (NRM-PZ P15827), TELM 4 (Ypresian, Early Eocene), one specimen from Jaw site (NRM-PZ P15828), TELM 4 (Ypresian, Early Eocene), eight specimens from below IAA 1/90 (NRM-PZ P15829), TELM 4 (Ypresian, Early Eocene), 26 specimens from NRM I (NRM-PZ P15861), TELM 4 (Ypresian, Early Eocene), seven specimens from *Cucullaea* I (NRM-PZ P15862), TELM 4 (Ypresian, Early Eocene), two specimens from IAA 1/11 (NRM-PZ P15867), TELM 4 (Ypresian, Early Eocene), four specimens from Punta Sergios (NRM-PZ P15879), TELM 4 (Ypresian, Early Eocene), five specimens from South of *Megalodon* Site (NRM-PZ P15891), TELM 4 (Ypresian, Early Eocene), one specimen from *Cucullaea* I (NRM-PZ P15892), one specimen from NRM 6 (NRM-PZ P15893), TELM 4 (Ypresian, Early Eocene).

 Twelve specimens from NRM 11, *Natica*-Horizon (NRM-PZ P15897), TELM 5 (Ypresian, Early Eocene), 13 specimens from NRM 10 (NRM-PZ P15889), TELM 5 (Ypresian, Early Eocene), 29 specimens from IAA 2/95 ‘Marsupial Site’ (NRM-PZ P15902), TELM 5 (Ypresian, Early Eocene), ten specimens from IAA 1/90, ‘Ungulate Site’ (NRM-PZ P15904), TELM 5 (Ypresian, Early Eocene), two specimens from *Cucullaea* intermedia *Psesphophorus*-Horizon (NRM-PZ P15905), TELM 5 (Ypresian, Early Eocene), two specimens from south of Marsupial site (NRM-PZ P15906), TELM 5 (Ypresian, Early Eocene), one specimen from IAA 1/95 (NRM-PZ P15911), TELM 5 (Ypresian, Early Eocene), two specimens from Shark Valley, *Natica*-Horizon (NRM-PZ P15912), TELM 5 (Ypresian, Early Eocene), one specimen from *Natica*-Horizon (NRM-PZ P15922), TELM 5 (Ypresian, Early Eocene), two specimens from Pass Site (NRM-PZ P15924), TELM 5 (Ypresian, Early Eocene), three specimens of *Natica*-Horizon (NRM-PZ P15925, TELM 5 (Ypresian, Early Eocene).

*Stratigraphic and geographic range.* TELM 2, Ypresian, Early Eocene: *Natica*-Lens, Channel Site.

TELM 4, Ypresian, Early Eocene: Shark basin, *Cucullaea* I, Jaw Site, below 1/90, NRM I (GPS data: 64°14.285′S, 56°40.182′W), *Cucullaea* I, IAA 1/1, Punta Sergios (GPS data: 64°14.168′S, 056°40.264′W), South of *Megalodon* Site, *Cucullaea* I and NRM 6.

TELM 5, Ypresian, Early Eocene: IAA 1/90 (GPS data: 64°14′04.67″S; 56°39′56.38″W), IAA 2/95, NRM 10, *Natica*-Horizon (GPS data: 64°13′950′S, 56°37′768′W), NRM 11, *Natica*-Horizon (GPS data: 64°13.849′S; 056°37.193′W), *Cucullaea* intermedia, south of Marsupial Site, IAA 1/95, Shark Valley, Pass site (GPS data: 64°14.144′S; 056°39.917′W), *Natica*-Horizon.

*Description.* In occlusal view, medial teeth are six-sided, and wider than long. The occlusal tooth surface is smooth and flat to slightly concave. The root exhibits the characteristic polyaulacorhize vascularization. Three different morphological types among the fossil teeth we studied can be distinguished.

Morphotype 1 (Figure 6(A–C)) is characterized by shorter root lobes than Morphotype 2. In most specimens the crowns are low (lower than crowns of Morphotype 2). Completely preserved specimens have straight to slightly arched crowns towards the middle part of the crown. In occlusal view, some teeth have a convex labial crown edge and a lingually concave crown edge. The labial and lingual crown faces are straight and strongly ornamented with vertical enameloid costules. Labially, the crown overhangs the root. Lingually, the root protrudes below the crown. The lingual ledge/bulge is prominent but not very thick. In profile view, the root is displaced lingually. Measurements of well-preserved specimens range from 18 mm to 47 mm in mesio-distal width and from 4 mm to 8 mm in labio-lingual length.

Morphotype 2 (Figure 6(D–E)) is characterized by high, narrow, and very prominent root lobes, when compared to the crown height. The crowns are significantly higher than in Morphotype 1 (considering abrasions). The occlusal surface is smooth, and slightly to strongly abraded, due to wear of the teeth. The labial and lingual crown faces are straight and are sculptured with deep vertical enameloid costules. The occlusal crown face is straight to slightly curved medially when viewed labially. In labial view, the crown overhangs the root. The root lobes are displaced lingually and protrude below the crown lingually.

The root lobes are very narrow and high. Labially, the root lobes are directed straight basally, lingually they protrude slightly below lingual ledge/bulge. This ledge/bulge is prominent but not as broad as in Morphotype 1. The crowns of well-preserved specimens differ in labio-lingual width ranging from 0.5 to 0.9 mm.

Morphotype 3 (Figure 6(F)) can be distinguished from the other two morphotypes by the prominent, elongated and slightly curved root lobes, the rather high crown with a completely straight occlusal surface.

*Remarks.* Isolated median and lateral myliobatiform teeth are rather common in the La Meseta and Submeseta formations, whereas complete or partially preserved tooth plates have not been recovered up to now. Fossil isolated teeth are often difficult to distinguish and to identify to species level, because there is a great variation in dental morphologies and unique characters are seemingly difficult to identify. Although, the highly variable dental morphology of pavement teeth of extant genera such as *Myliobatis* Cuvier (ex Dumeril) 1816, *Rhinoptera* Cuvier 1829; *Aetomylaeus* Garman 1908 *; Aetobatus* Blainville, 1816 renders the identification of isolated teeth extremely difficult (Nishida 1990; Herman et al. 2000; Cappetta 2012). Most authors provide different characteristics to distinguish between different taxa of myliobatiform species (e.g. height of the crown, ornamentation of the labial and lingual crown faces, the appearance of the root, the number of root lobes and their width). However, these differences are challenging to recognize in isolated fossil teeth, which are not well preserved or fragmented. Furthermore, teeth of the genus *Myliobatis* show a significant range of morphological variations, which can be explained by the heterodonty pattern of medial, lateral and posterior files.

Comparing the six figured specimens in Figure 6(A–F) it is obvious that in the Eocene of Antarctica three different morphotypes of myliobatid teeth are present, but most of the teeth are fragmented, worn or abraded and their taxonomic identification is rather difficult. The low crowned specimens shown in Figure 6(A–C) might represent specimens assignable to *Myliobatis*, because they are characterized by rather low crowns, hexagonal occlusal surfaces, which are more or less straight, strong ornamentation on the labial and lingual crown faces and the more numerous and narrow root lobes. The high-crowned specimens (e.g. Figure 6(D–E)) could represent both *Myliobatis* and *Rhinoptera*, but due to their incomplete and fragmented nature we are not certain about their correct placement. The last figured specimen (Figure 6(F)) resembles teeth of *Aetobatus*, but differs significantly in the height of the crown. The crown is much higher in the Antarctic specimen described here compared to other fossil *Aetobatus* teeth and the root lobes are shorter in labial view (e.g. Antunes and Balbino 2006; Maisch et al. 2014; Malyshkina and Ward 2016; Ebersole et al. 2017). Hovestadt and Hovestadt-Euler (2013) questioned whether any of the fossil members of the genus *Aetobatus* can truly be assigned, because of the individual variation within the tooth plates of extant *Aetobatus* species. However, their incomplete nature as it is the case for most Antarctic specimens as well as the lack of associated complete tooth plates, makes any determination at species level rather difficult. Only the study of extant species can help to solve this problem.

## Discussion

**Figure 7.**
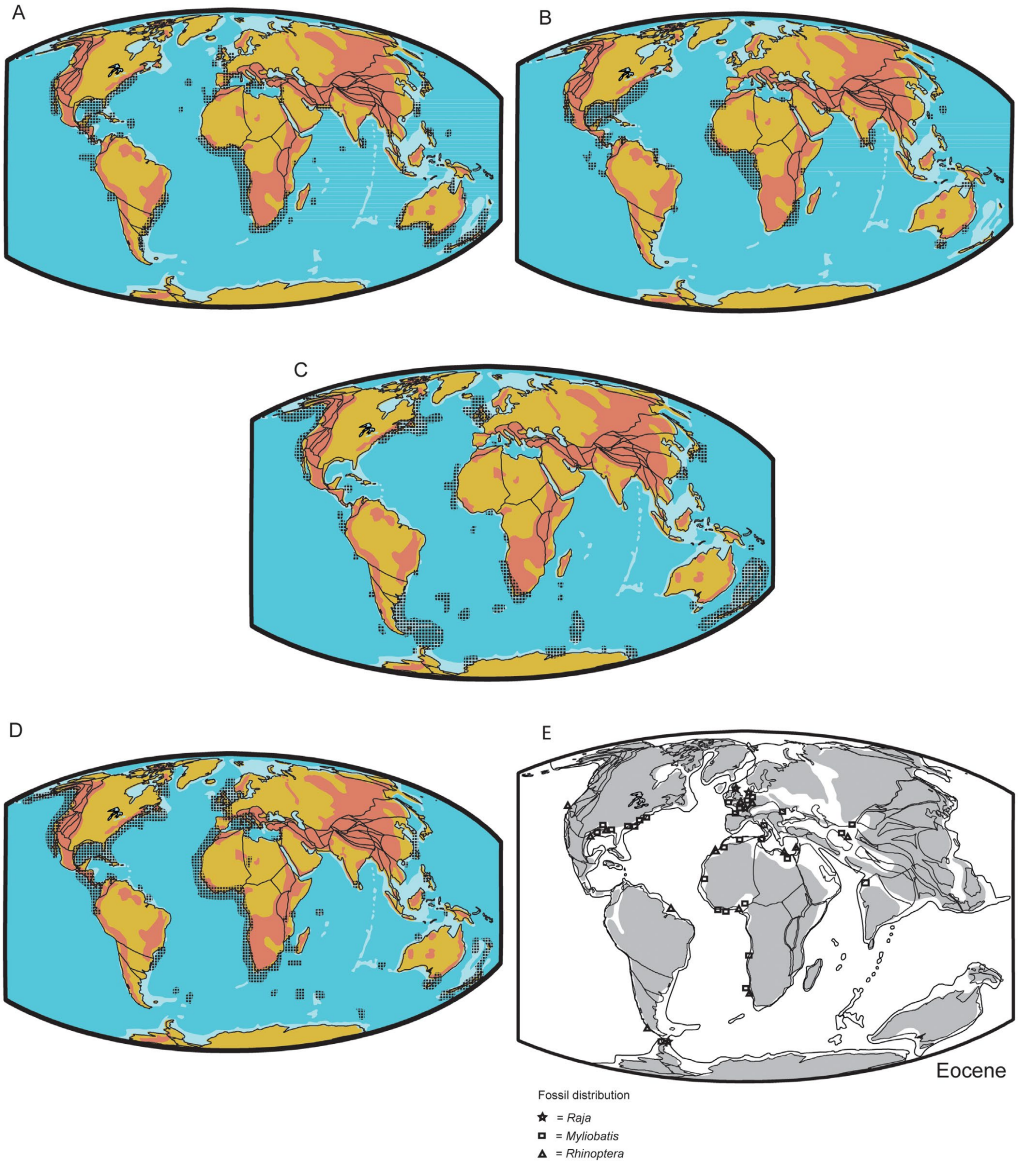
Distribution of extinct and extant species of the genera *Rhinoptera*, *Myliobatis* and *Raja*. The extinct taxa are marked with symbols. (A), Geographic distribution of extant *Myliobatis* spp; (B), Geographic distribution of extant *Rhinoptera* spp.; (C), Geographic distribution of extant *Bathyraja* ssp.; (D), Geographic distribution of extant *Raja* spp.; (E), Paleogeographic distribution of fossil remains of the genera *Rhinoptera*, *Myliobatis* and *Raja*.

A highly endemic fish fauna characterizes modern Antarctic waters where osteichthyans are predominantly represented by notothenoid fishes (icefishes), and skates (Rajidae) are the dominant chondrichthyans, today. The nine skate species known to inhabit the Southern Ocean, *Amblyraja georgina* Norman, 1938; *Amblyraja taaf* Meissner, 1987; *Bathyraja meridionalis* Stehman, 1987; *Bathyraja eatonii* Günther, 1876; *Bathyraja irrasa* Hureau and Ozouf-Costaz, 1980 and *Bathyraja murray* Günther, 1880; are also found in the South Atlantic and Indian Ocean, whereas *Bathyraja maccaini* Springer, 1971; *Bathyraja* sp. (cf *eatonii*) and *Bathyraja* sp. (dwarf) are circum-polarly distributed (Amsler et al. 2015). Sharks, like *Lamna nasus* Bonnaterre, 1788; the porbeagle shark, are recorded only occasionally in the Southern Ocean. According to Long (1994) skates are the only survivors of a highly diversified cold water-adapted chondrichthyan fauna from the Eocene of the La Meseta Formation. However, rays and skates are only a minor component in Antarctic Eocene strata compared to the overwhelming amount of selachians, which account for up to 93% of all teeth that have been recovered thus far. Selachians and batoids are most abundant in TELMs 4 and 5 (Ypresian, Early Eocene). Species richness of the chondrichthyan fauna in the La Meseta Formation increases from TELM 1 to TELM 5 but decreases from TELM 5 onwards (Kriwet et al. 2016). Estimated sea-surface temperatures for TELM 3 to TELM 6 are moderate to cool, with temperatures reaching 15 °C in TELM 3 and dropping to 7 °C by the end of TELM 6 (middle Eocene). The depositional environments for these intervals is purportedly deposited in deltaic to estuarine (see Kriwet et al. 2016). All myliobatiform teeth come from this time interval more specific from TELMs 2, 4 and 5. No myliobatiform teeth have been thus far found in the lowest and the uppermost parts (TELMs 1, 3 and 7) of the section when temperatures were below 10 °C. Most of the myliobatiform remains were found in TELM 5, which is Ypresian (Early Eocene) in age. Conversely, rajid teeth were only collected from TELMs 5 and 6 (through bulk-sampling). The absence of batoids from the lowermost and uppermost parts of the La Meseta and Submeseta formations might represent collection biases especially for rajid teeth, because bulk-sampling was conducted only in selected layers. Additionally, the water temperatures recorded for TELM 7 might already have been too cold for myliobatids, as modern representatives are most abundant in temperate to tropical waters (Figure 7(A–B)).

Examining the global Eocene fossil record (Figure 7(E)), batoids seem to have been more abundant in the Northern than in the Southern Hemisphere. Fossil remains of Myliobatiformes are known from many localities in marine and freshwater deposits (de Carvalho et al. 2004). Isolated teeth and dermal denticles are the most abundant remains, whereas more complete or articulated specimens are rare in the fossil record, and are known from few localities, like the Monte Bolca Formation (marine) of north-eastern Italy, and the Green River Formation (freshwater) of Wyoming, both of which are of Early Eocene age (de Carvalho et al. 2004). Eocene elasmobranch assemblages of Europe, Africa and North America are characterized by a high diversity of myliobatiform rays (e.g. Noubhani and Cappetta 1997; Adnet et al. 2010; Claeson et al. 2010; Underwood et al. 2011; Cappetta 2012; Clayton et al. 2013; Carlsen and Cuny 2014; Carnevale et al. 2014; Cappetta and Case 2016).

In contrast to myliobatids, rajid remains are rather rare in the fossil record but the group is very widespread in modern seas (Figure 7(C–E)). The earliest records of fossil rajids are known from the lower Cenomanian of Lebanon (e.g. Davies 1888; Hückel 1970; Cappetta 1980; Underwood 2006). Fossil teeth of rajids are more commonly found in Cenozoic than in Late Cretaceous deposits (Cappetta 1987). The identification of supposed Late Cretaceous remains of *Raja* still needs to be verified. In the Eocene, European representatives of the genus *Raja* are only known by isolated teeth. In contrast, the genus *Bathyraja*, which is one of the two extant families, that have successfully colonized the Southern Ocean, has no verified fossil record.

## Conclusions

Skates and rays are an uncommon group within the very fossiliferous Eocene deposits of the La Meseta and Submeseta formations, which contrasts with the abundant fossil remains of sharks, that had been found and described to date (e.g. Long 1992a, 1992b; Cione and Reguero 1994, 1998; Welton and Zinsmeister 1980; Kriwet 2005; Kriwet et al. 2016; Engelbrecht et al. 2016a, 2016b). The rare finds of skate teeth in these sediments provide a minimum age constraint to the Ypresian (Early Eocene) for their first appearance in the Southern Ocean. These finds indicate that in the Early Eocene the temperature and the environmental settings of the Southern Ocean were favourable for them. Furthermore, the material allows us to established two new genera, *Marambioraja* gen. nov. and *Mesetaraja* gen. nov, and two new species within the genus *Raja*, *R. amphitrita* sp. nov and *R. manitaria* sp. nov. The rajid remains previously described by (Long 1994) are assigned *Raja manitaria* sp. nov. due to their strong morphological resemblance.

## Disclosure statement

No potential conflict of interest was reported by the authors.

## Funding

This work was supported by The Austrian Science Fund [FWF, grant number P26465-825] to J.K.; a graduation scholarship of the University of Vienna to A.E.; the Swedish Research Council [VR grant number 2009-4447] to T.M.; the Consejo Nacional de lnvesligaciones Cienlificas y Tecnicas (CONICET) [grant number PIP 0462] to M.R.; and the Argentinian National Agency for Promotion of Science and Technology (ANPCyT) [grant number PICTO 0093/2010] to M.R.
